# Research Advances in Multi-Omics on the Traditional Chinese Herb *Dendrobium officinale*

**DOI:** 10.3389/fpls.2021.808228

**Published:** 2022-01-11

**Authors:** Yue Wang, Yan Tong, Oluwaniyi Isaiah Adejobi, Yuhua Wang, Aizhong Liu

**Affiliations:** ^1^Key Laboratory of Economic Plants and Biotechnology, Yunnan Key Laboratory for Wild Plant Resources, Kunming Institute of Botany, Chinese Academy of Sciences, Kunming, China; ^2^Bio-Innovation Center of DR PLANT, Kunming Institute of Botany, Chinese Academy of Sciences, Kunming, China; ^3^Key Laboratory for Forest Resources Conservation and Utilization in the Southwest Mountains of China, Ministry of Education, Southwest Forestry University, Kunming, China

**Keywords:** *Dendrobium officinale*, multi-omics, bioactive compounds, biosynthesis, regulation

## Abstract

*Dendrobium officinale* Kimura et Migo is an important epiphytic plant, belonging to the Orchidaceae family. There are various bioactive components in *D. officinal*e plants, mainly including polysaccharides, alkaloids, and phenolic compounds. These compounds have been demonstrated to possess multiple functions, such as anti-oxidation, immune regulation, and anti-cancer. Due to serious shortages of wild resources, deterioration of cultivated germplasm and the unstable quality of *D. officinale*, the study has been focused on the biosynthetic pathway and regulation mechanisms of bioactive compounds. In recent years, with rapid developments in detection technologies and analysis tools, omics research including genomics, transcriptomics, proteomics and metabolomics have all been widely applied in various medicinal plants, including *D. officinale*. Many important advances have been achieved in *D. officinale* research, such as chromosome-level reference genome assembly and the identification of key genes involved in the biosynthesis of active components. In this review, we summarize the latest research advances in *D. officinal*e based on multiple omics studies. At the same time, we discuss limitations of the current research. Finally, we put forward prospective topics in need of further study on *D. officinale*.

## Introduction

*Dendrobium officinale* Kimura et Migo, an important epiphytic plant in the Orchidaceae family, has been used as a traditional Chinese medicine herb for thousands of years in China, and widely as a medicinal herb in many Asian countries (Ng et al., 2012). Wild *D. officinale* is often distributed in tropical regions, and it has been widely introduced to subtropical regions for cultivation. Generally, *D. officinale* can nourish the stomach, moisten the lung, relieve coughs and strengthen the body (Ng et al., 2012; Tang et al., 2017; Chen W. et al., 2021). Chemical isolation of compounds has identified diverse active compounds including polysaccharides, flavonoids, alkaloids, and multiple amino acids. In particular, the polysaccharides, alkaloids, and flavonoids extracted from *D. officinale* are thought to be the main bioactive ingredients for medicine (Meng et al., 2016; Tang et al., 2017; Wang et al., 2020; Wang, 2021). Pharmacological studies have confirmed the positive effects of polysaccharides, alkaloids and flavonoids extracted from *D. officinale* on immune regulation and liver protection (Wang et al., 2020), on anti-cancer and neuroprotection (Zhang et al., 2003; Wang et al., 2020), and on anti-oxidant activity (Yuan et al., 2020a). Phenols extracted from *D. officinale* (such as erianin and gigantol) are active in enhancing immune activity while also exhibiting anti-tumor and anti-oxidant properties (Zhang et al., 2018; He et al., 2020). Owning to the increasing demands for diverse healthy products based on *D. officinale*, the cultivation of *D. officinale* is expanded throughout south China and south Asian countries with an annual output value of 10 billion Yuan per year (Wang and Shi, 2019).

Studies have revealed that the content of active compounds often varies among different tissues in *D. officinale*. For instance, stems accumulate the most abundant polysaccharides (Wang et al., 2020; Yuan et al., 2020a), while leaves contain higher contents of flavonoids and alkaloids than other tissues (Shen et al., 2017; Chen et al., 2019; Yuan et al., 2020b; Wang, 2021). Bibenzyls (belonging to phenolic compounds) including erianin and gigantol are mainly detected in roots and stems (Adejobi et al., 2021). Moreover, increasing evidence shows that the biosynthesis and accumulation of diverse active compounds are often influenced by various environmental factors such as different cultivation substrates (Zuo et al., 2020) and habitats (Lei et al., 2018). Therefore, it is important to dissect the physiological and molecular mechanisms underlying accumulation of those active compounds during the growth and development of different tissues for breeding by genetic improvement, cultivation and management practices in agriculture. In the last decade, technical development of DNA sequencing and molecular identification has led to the extensive application of diverse omics such as genomics, transcriptomics, proteomics and metabolomics, which are used to dissect the physiological and molecular mechanisms of active compound biosynthesis. These multi-omics studies have provided vital insights into the understanding of the biosynthesis pathways of active compounds and their molecular regulation networks during the growth and development of *D. officinale*. In this review, we focus on the genetic and molecular bases of active compounds identified from *D. officinale* based on the latest research on multi-omics in *D. officinale* to aid more studies in the discovery of the physiological and molecular mechanisms that underlie those active compounds accumulated in *D. officinale*, facilitating directional breeding, cultivation and management, and medicinal usage.

## Genomic Research in *Dendrobium Officinale*

Genome data can provide basic and comprehensive information on genetic backgrounds and bridge gaps between genetic bases and active compounds in medicinal plants. To date, three versions of *D. officinale* genomic data (2n = 38) have been reported, with the genome size varying from 1.01 to 1.36 Gb and heterozygosity of 0.48–1.27% (Yan et al., 2015; Zhang G. Q. et al., 2016; Niu et al., 2021). A comparison between them is listed in Table 1. The first two versions were assembled at the scaffold level, while the third version was assembled onto 19 chromosomes with third-generation and Hi-C sequencing platforms (Niu et al., 2021). The number of protein-coding genes varied from 27631 to 35567, with 629–1462 gene families specific to *D. officinale*. There are differences in genomic data between three versions, which could result from the sequencing platform, assembly strategies and genome annotation methods. The genome of *D. officinale* characteristically features abundant repetitive sequences and two rounds of whole-genome duplication (WGD; Zhang G. Q. et al., 2016; Niu et al., 2021). In the future, with the aid of genetic and physical maps and more transcriptomic data, genome quality and annotation integrity can be further improved.

**TABLE 1:** A quality comparison of three *D. officinale* genome versions.

Items	v.1.0 (Yan et al., 2015)	v.2.0 (Zhang G. Q. et al., 2016)	v.3.0 (Niu et al., 2021)
Sequencing platform	Illumina HiSeq 2000 PacBio RS II	Illumina HiSeq 2000	Illumina HiSeq 2500 PacBio Sequel II
Genome size	1.36 Gb	1.01 Gb	1.23 Gb
Heterozygosity	0.48%	0.628%	1.27%
Contig N50	25.12 kb	33.09 kb	1.44 Mb
Contig number	814,881	105,732	2,430
Scaffold N50	76.49 kb	391.46 kb	63.07 Mb
Assembly level	Scaffold	Scaffold	Chromosome
WGD	–	2	2
Repetitive ratio	63.33%	78.1%	64.39%
SNPs	5,432,657	5,758,781	–
Protein-coding genes	35,567	28,910	27,631
Specific gene families	1,462	629	1,196
Functionally annotated genes	34,699	–	25,894

**FIGURE 1:**
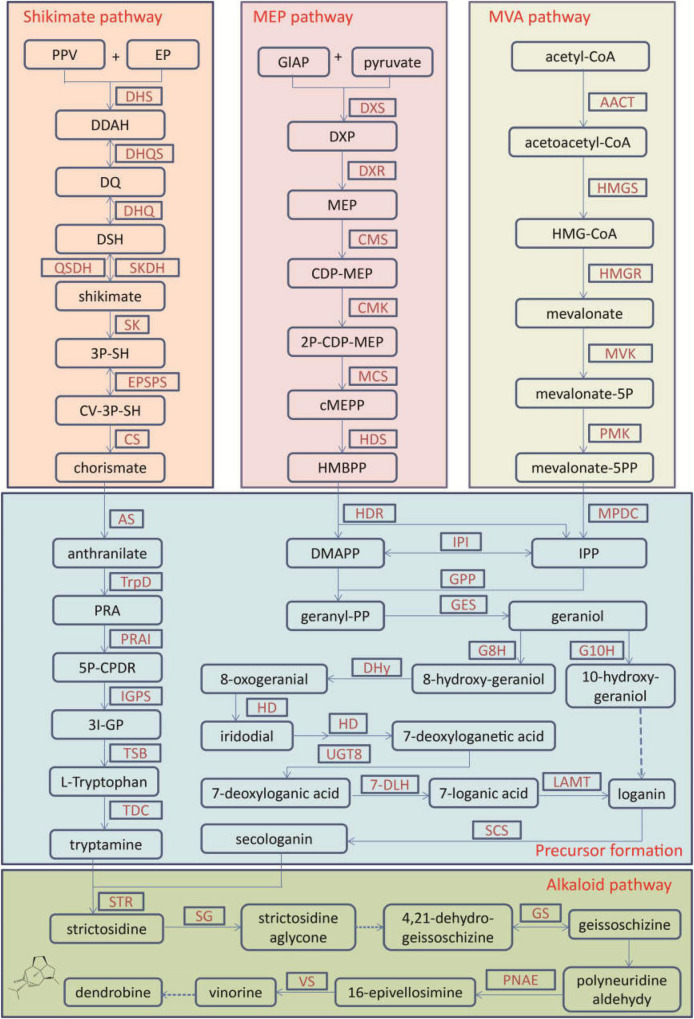
The biosynthetic pathway for terpenoid indole alkaloids in *Dendrobium officinale*. The alkaloid biosynthesis contains the precursor formation from the shikimate, MEP, and MVA pathways and the alkaloid pathway. 2P-CDP-MEP, 2-phospho-4-(cytidine 5′-diphospho)-2-C-methyl-D-erythritol; 3I-GP, (3-indoyl)-glycerolphosphate; 3P-SH, shikimate 3-phosphate; 5P-CPDRC, 1-(2-carboxyphenylamino)-1′-deoxy-D-ribulose 5-phosphate; CDP-MEP, 4-(cytidine 5′-diphoshpo)-2-C-methyl-D-erythritol; cMEPP, 2-C-methyl-D-erythritol-2,4-cyclodiphosphate; CV-5P-SH, 5-O-(1-carboxyvinyl)-3-phosphoshikimate; DDAH, 7P-2-dehydro-3-deoxy-D-arabino-heptonate; DMAPP, dimethylallyl-PP; DQ, 3-dehydroquinate; DSH, 3-dehydroshikimate; DXP, 1-deoxy-D-xylulose-5-phosphate; EP, D-erythrose 4-phosphate; HMBPP, 1-hydroxy-2-methyl-2-butenyl-4-diphosphate; HMG-CoA, 3-hydroxy-3-methylglutaryl-CoA; IPP, isopentenyl-PP; MEP, 2-C-methyl-D-erythritol-4-phosphate; PPV, phosphoenolpyruvate; PRA, N-(5-phospho-β-D-ribosyl)-anthranilate; 7-DLH, 7-deoxyloganate 7-hydroxylase; AACT, acetyl-CoA acetyltransferase; AS, anthranilate synthase; CMK, 4-diphosphocytidyl-2C-methyl-D-erythritol kinase; CMS, 4-diphosphocytidyl-2C-methyl-D-erythritol synthase; CPR, NADPH-hemoprotein reductase; CS, chorismate synthase; DHQ, 3-dehydroquinate dehydratase; DHQS, 3-dehydroquinate synthase; DHS, 3-deoxy-7-phosphoheptulonate synthase; DHy, dehydrogenase; DXR, 1-deoxy-D-xylulose-5-phosphate reductoisomerase; DXS, 1-deoxyxylulose-5-phosphate synthetase; EPSPS, 3-phosphoshikimate 1-carboxyvinyltransferase; G10H, geraniol 10-hydroxylase; G8H, geraniol 8-hydroxylase; GPP, geranyl diphosphate diphosphatase; GES, geranyl diphosphate synthase; GS, geissoschizine synthase; HD, hydroxylase; HDR, 4-hydroxy-3-methylbut-2-en-1-yl diphosphate reductase; HDS, 1-hydroxy-2-methyl-2-(E)-butenyl-4- diphosphate synthase; HMGR, HMG-CoA reductase; HMGS, HMG-CoA synthase; IGPS, indole-3-glycerolphosphate synthase; IPI, IPP isomerase; LAMT, loganate O-methyltransferase; MCS, 2-C-methyl-D-erythritol 2,4- cyclodiphosphate synthase; MPDC, mevalonate diphosphate decarboxylase; MVK, mevalonate kinase; PMK, phosphomevalonate kinase; PNAE, polyneuridine-aldehyde esterase; PRAI, phosphoribosylanthranilate isomerase; QSDH, quinate/shikimate dehydrogenase; SCS, secologanin synthase; SG, strictosidine β-D-glucosidase; SK, shikimate kinase; SKDH, shikimate dehydrogenase; STR, strictosidine synthase; TDC, tryptophan decarboxylase; TrpD, anthranilate phosphoribosyltransferase; TSB, tryptophan synthase; UGT8, 7-deoxyloganetic acid glucosyltransferase; VS, vinorine synthase.

**FIGURE 2:**
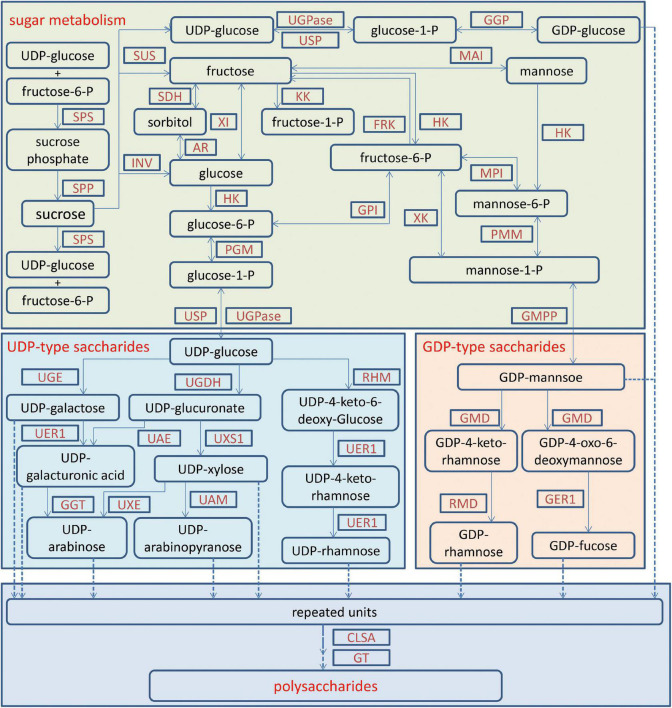
The biosynthetic pathway for polysaccharides in *Dendrobium officinale*. The biosynthesis of polysaccharides contains sugar metabolism, UDP-saccharide metabolism, GDP-saccharide metabolism and polysaccharide formation. AR, aldose reductase; CSLA, cellulose synthase-like A; FRK, fructokinase; GAE, UDP-glucose A-4-epimerase; GER1, GDP-4-keto-6-deoxy-D-mannose-3,5-epimerase-4-reductase; GGP, glucose-1-phosphate guanylyltransferase; GGT, UDP-galacturonate decarboxylase; GMD, GDP-D-mannose-4,6-dehydratase; GMPP, mannose-1-phosphate guanylyl transferase; GPI, glucose-6-phosphate isomerase; GT, glycosyltransferases; HK, hexokinase; INV, invertase; KK, ketohexokinase; MAI, mannose isomerase; MPI, mannose-6-phosphate isomerase; PGM, phosphoglucomutase; PMM, phosphomannomutase; RHM, UDP-glucose 4, 6-dehydratase; RMD, GDP-4-dehydro-D-rhamnose reductase; SDH, sorbitol dehydrogenase; SPP, sucrose phosphate phosphatase; SPS, sucrose phosphate synthase; SuS, sucrose synthase; TSTA3, GDP-l-fucose synthase; UAE, UDP-glucuronate 4-epimerase; UAM, UDP-arabinopyranose mutase; UER1, 3,5-epimerase/4-reductase; UGDH, UDP-glucose dehydrogenase; UGE, UDP-glucuronate 4-epimerase; UGPase, UDP-glucose pyrophosphorylase; USP, UDP-sugar pyrophosphorylase; UXE, UDP-arabinose 4-epimerase; UXS1, UDP-glucuronate decarboxylase; XI, xylose isomerase; XK, xylulokinase.

**FIGURE 3:**
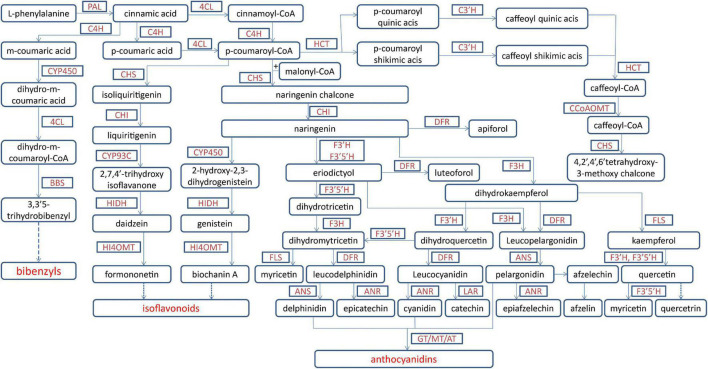
The biosynthetic pathway for phenols in *Dendrobium officinale*. The biosynthesis for main phenols includes anthocyanidins, isoflavonoids, flavones, flavonols and bibenzyls from phenylalanine. 4CL, 4-coumarate-CoA ligase; ANR, anthocyanidin reductase; ANS, anthocyanidin synthase; AT, acyltransferase; BBS, bibenzyl synthase; C3′H, 5-O-(4-coumaroyl)-D-quinate 3′-monooxygenase; C4H, cinnamate 4-hydroxylase; CCoAOMT, caffeoyl-CoA O-methyltransferase; CHI, chalcone isomerase; CHS, chalcone synthase; CYP450, cytochrome P450-dependent monooxygenase; DFR, dihydroflavonol-4-reductase; F3′5′H, flavonoid 3′,5′-hydroxylase; F3′H, flavonoid 3′-monooxygenase; F3H, flavonoid 3-hydroxylase; FLS, flavonol synthase; GT, glycosyltransferase; HCT, quinate O-hydroxycinnamoyltransferase; HI4OMT, hydroxy-isoflavanone-4- O-methyltransferases; HIDH, tri-hydroxy-isoflavanone dehydratase; LAR, leucoanthocyanidin dioxygenase; MT, methyltransferase; PAL, phenylalanine ammonia lyase.

For medicinal plants, one of the most important studies in genomics is to annotate and identify candidate genes related to the biosynthesis of active compounds. Three versions of the *D. officinale* genome showed marked expansion of some genes encoding the enzymes responsible for the biosynthesis of active compounds. According to the earlier two versions, some key enzyme genes involved in the biosynthesis of active compounds were identified (Yan et al., 2015; Zhang G. Q. et al., 2016). Based on the third version, the researchers comprehensively identified the genes in the biosynthetic pathways of polysaccharides, alkaloids, and flavonoids, including 418 genes (Niu et al., 2021; Figures 1–3). For the biosynthesis of polysaccharides, a total of 268 genes encoding 56 enzymes were identified (Niu et al., 2021). Among them, there are 13, 10, and 15 genes that encoded cellulose synthase-like A (CLSA), sucrose-phosphate synthase and sucrose synthase, respectively, which showed expansion (Yan et al., 2015; Zhang G. Q. et al., 2016). In particular, the galacturonosyltransferase and β-galactosidase gene families are uniquely evolved in *D. officinale* (Yan et al., 2015). The large-scale expansion of these genes may partly explain the abundant polysaccharides in *D. officinale*. For the two other types of active compounds (alkaloids and flavonoids), there are 98 and 52 genes that could be involved in the biosynthesis pathways, respectively (Niu et al., 2021). A putative enzyme (polyneuridine-aldehyde esterase) may be responsible for the extension of 16-epivellosimine (Yan et al., 2015; Figure 1). Moreover, other genes related to distinctive traits were also annotated and identified, including fungus symbiosis, drought resistance, photosynthesis, and flower development (Yan et al., 2015; Zhang G. Q. et al., 2016; Niu et al., 2021). The analysis of those genes suggests their adaptation to certain environments.

Moreover, *D. officinale* has distinct flower architecture, which has attracted researchers’ attention. Genomic research provides important insights on flower development in *D. officinale*. The ABCDE model is a classical model for flower architecture. Although there are fewer MADS-box genes (63) in *D. officinale* than that in rice and Arabidopsis, five classes of MADS-box genes all exist, suggesting presence of complete flower genes in *D. officinale* (Yan et al., 2015; Zhang G. Q. et al., 2016). Unique genes, gene expansion and gene loss were observed in MADS-box gene family. Among them, MADS-box ZMM17 gene family, belong to B class, is unique in *D. officinale*, which could be related to the distinct flower architecture (Yan et al., 2015). ANR1, StMADS11, and MIKC* genes generally participate in regulation of growth and development, and their expansion could result in the diversity (Zhang G. Q. et al., 2016). The less number of type I MADS box genes may lead to the failure of endosperm development (Zhang G. Q. et al., 2016). The genomic investigation provides important hues for understanding the distinct and various flowers in *D. officinale*.

The genomic research of *D. officinale* provides abundant information for understanding the potential genetic resources of active component accumulation and insights for some important traits. In the future, confirming the key genes related to the above traits will be a research focus, and Genome-Wide Association Studies (GWAS) could be one of the principle methods for mapping candidate genes.

## Transcriptomic Research in *Dendrobium Officinale*

Transcriptomic sequencing (RNA-seq) has been widely applied to research in medicinal plants including *D. officinale* due to low expenditure, high throughout, high sensitivity and no limitation from genome sequencing requirement. Since the first transcriptome of *D. officinale* stems was reported (Guo et al., 2013), there are now up to 30 transcriptome data of *D. officinale* available in the NCBI (Table 2). This research characterizes the biosynthetic pathways of active compounds in *D. officinale*, which provides an important basis for its uses as a medicinal material (Table 1; Yuan et al., 2020a). Transcriptomic analysis was also used to investigate the effects of various factors on the accumulation of active compounds, including the material location, hormones and more (Chen et al., 2019; Ren et al., 2020; Zuo et al., 2020). Moreover, the potential molecular mechanisms of a variety of physiological processes were analyzed, such as response to stress, flowering and symbiotic relationships (Wang et al., 2018; Zou et al., 2018; Li H. et al., 2021).

**TABLE 2:** A summary of transcriptomic reports in *D. officinale.*

Tissues	Treatments or conditions	Research focus	References
S	–	Alkaloid, genetic markers	Guo et al., 2013
F, R, L, and S	–	Organ-specific study	Meng et al., 2016
L, S, R, and F	–	Polysaccharide synthesis, alkaloid synthesis	Shen et al., 2017
S and L	–	Variant splicing, sugar translocation	He et al., 2017
Eight tissues	–	Expression	Zhang et al., 2017
R, S, and L	–	Flavonoid biosynthesis	Yuan et al., 2020b
L, S, R, and F	–	Flowering	Chen Y. et al., 2017
L, R, S, and F	–	Polysaccharide synthesis	Zhang G. Q. et al., 2016
PLB, L	–	Alkaloid synthesis	Wang et al., 2021
Seedlings	Juvenile and adult	Polysaccharide synthesis	Zhang J. et al., 2016
S	Four stages	Mannan polysaccharides	He et al., 2015
R	Cadmium stress	Stress response	Jiang et al., 2020
L	MeJA	Accumulation of alkaloids	Chen et al., 2019
Seedlings	Far-red light	Shade-avoidance	Li H. et al., 2021
Seeds	Symbiotic and asymbiotic	Germination	Chen J. et al., 2020
L	Drought	Drought stress	Wan et al., 2018
L	CO_2_	Crassulacean acid metabolism	Zou et al., 2018
Germinated seeds	Symbiotic and asymbiotic seeds	Symbiotic association	Wang et al., 2018
L	Drought stress and stress removal	Drought stress	Zou et al., 2018
Plants	SA	Polysaccharide, flavonoid, and alkaloid synthesis	Niu et al., 2021
R	MeJA	Bibenzyl biosynthesis	Adejobi et al., 2021
S	Three provinces	Flavonoids	Lei et al., 2018
S	Three provinces	Anthocyanins	Ren et al., 2020
S, L, and R	Three species	Polysaccharide and alkaloid synthesis	Yuan et al., 2020a
Styles	Non-, self-, and cross-pollinated	Self-incompatibility	Chen Y. et al., 2021

*S, stems; L, leaves; F, flowers; R, roots; PLB, protocorm-like bodies; Eight tissues – L, S, column, flower buds, lip, sepal, white root, and green root tip.*

Tissue-specifically expressed genes can be identified through analysis of comparative transcriptomics. A total of 2645, 256, 42, and 54 transcripts were highly expressed in four organs (flower, root, leaf, and stem), respectively (Meng et al., 2016). Those differential expression genes (DEGs) were shown to be involved in organ-specific functions. Among them, eight genes with high transcripts in stem were annotated to the secondary metabolic pathways and carbohydrate transport and metabolism, which was consistent with its abundant metabolites. Moreover, 25 genes were identified to participate in the regulation of flowering, which suggests the presences of classical flowering pathways (Shen et al., 2017).

Alkaloids are one type of the earliest-identified active compounds in *D. officinale* (Tang et al., 2017). Although there are different types of alkaloids accumulated in *D. officinale* (Jiao et al., 2018), the main classes of alkaloids are terpenoid indole alkaloids (TIAs; Guo et al., 2013; Niu et al., 2021). Alkaloids are present in multiple tissues, including protocorm-like bodies (PLB), flower, root, leaf and stem, and the content in PLBs was the highest (Wang et al., 2021). There were 25 genes that participated in the shikimate, mevalonate (MVA) and methylerythritol phosphate (MEP) and strictosidine pathways, and other genes were potentially related to the alkaloid synthesis, such as cytochrome P450s, aminotransferases, methyltransferases, multidrug resistance protein (MDR), transporters and transcription factors (Guo et al., 2013; Figure 1). Further expression analysis confirmed that the genes responsible for 1-deoxy-D-xylulose-5-phosphate synthase (DXS), 5-enolpyruvylshikimate-3-phosphate synthase, shikimate dehydrogenase, mevalonate kinase (MVK), and aminotransferases were mainly expressed in leaves, which could be the main reason for higher alkaloid content in leaves (Shen et al., 2017). Recently, 41 genes showing significant differences in expression levels were detected between PLBs and leaves, including genes in the strictosidine biosynthesis (Wang et al., 2021). The candidate genes encoding strictosidine β-D-Glucosidase, geissoschizine synthase and vinorine synthase in the alkaloid biosynthesis enzymes were first identified, which facilitates the analysis of its metabolite biosynthesis in future studies.

Polysaccharides in *D. officinale* are the most abundant active compounds in various tissues and adult stems show the highest content (He et al., 2015; Zhang J. et al., 2016; Shen et al., 2017). A metabolic pathway for fructose and mannose biosynthesis has been developed through comparative transcriptomic analysis of different tissues, including 44 genes (He et al., 2015; Figure 2). The expression patterns of eight *CELLULOSE SYNTHASE*-*LIKE A* (*CSL*A) genes were closely linked to the content of mannose in stems (He et al., 2015). Similarly, other putative genes responsible for higher polysaccharides in adult stems were identified, including mannose-1-phosphate guanylyltransferase, GDP-L-fucose synthase and mannose-6-phosphate isomerase (Shen et al., 2017). Using the PacBio sequencing technique, alternative splicing forms of two glycosyltransferase and four cellulose synthase genes have been detected, and two genes encoding SWEET and sucrose transporter showed higher expression in stems than in leaves (He et al., 2017). Although the biosynthesis for polysaccharides is complex, these studies provide hints for further investigation on the molecular mechanism of rich polysaccharides in *D. officinale*.

There are abundant phenols in *D. officinale*. Among them, flavonoids were found in leaves, stems, and roots of *D. officinale*, and the content in leaves was the highest (Yuan et al., 2020b). The biosynthetic pathway of flavonoids was proposed and contained 26 genes including chalcone synthase (CHS), flavanone 3-dioxygenase (F3H), dihydroflavonol reductase (DFR), flavonol synthase (FLS), *trans-*cinnamate 4-monooxygenase, leucoanthocyanidin dioxygenase (LAR), anthocyanidin reductase (ANR), and shikimate O-hydroxycinnamoyl transferase (Yuan et al., 2020b; Figure 3). DEGs were enriched in phenylpropanoid and flavonoid biosynthesis. Multiple structural genes in the flavonoid pathway were significantly upregulated in leaves, such as one *LAR1*, one *DFR3*, one *F3H*, and three *CHS* genes, which facilitate the accumulation of flavonoids in leaves and could be the main reason for different flavonoid contents among tissues. Recently, it has been reported that the contents of flavonoids and anthocyanins were different in the stems of *D. officinale* from three provinces (Lei et al., 2018; Ren et al., 2020). Different anthocyanin contents could result from the different activities of some key enzymes, such as hydroxyl cinnamomum acyltransferase 1, UDP-glycosyltransferase (UGT)-83A1, chalcone flavone isomerase-like, and UGT-88B1 serine carboxypeptidase-like 1 (Ren et al., 2020).

Exogenous application of plant hormones can induce accumulation of metabolites in medicinal plants, including in *D. officinale*. Treatments with methyl jasmonate (MeJA) and salicylic acid (SA) resulted in more active compounds accumulated in various tissues (Jiao et al., 2018; Chen et al., 2019; Adejobi et al., 2021; Niu et al., 2021). After MeJA treatment the alkaloids were enhanced in leaves of *D. officinale* through upregulating expression levels of the multiple genes in the pathways of MVA and MEP, such as P450 genes, transaminase genes and methyltransferase (Chen et al., 2019). The bibenzyl compounds were mainly accumulated in the roots and greatly increased after MeJA treatment because some key genes involved in the flavonoid and bibenzyl pathways were abundantly expressed after treatment, including P450 and putative bibenzyl synthase genes (Adejobi et al., 2021; Figure 3). Presence of SA in the culture medium can increase the accumulation of active components in *D. officinale* seedlings including alkaloids, polysaccharides, and flavonoids (Niu et al., 2021). As many as 107 genes involved in biosynthesis of active components were upregulated. Among them, two key enzyme genes (*F3*′*H* and *DFR*) involved in the anthocyanin synthesis were significantly upregulated, which may result in color differences between individual plants (Niu et al., 2021). These studies could help us improve the contents of bioactive compounds and supply information for further functional investigation of putative genes.

Transcriptomics can also be used to analyze other physiological processes. In nature *D. officinale* generally forms few seeds potentially because of self-incompatibility. A total of 41 putative genes involved in self-incompatibility were identified, including six Ca^2+^ signal genes and 11 S-locus receptor kinase (SRK) related genes (Chen Y. et al., 2021), which supply helpful insights for genetic mechanisms and preservation of *D. officinale* resources. Moreover, *D. officinale* seeds are small and have limited energy reserves, and the symbiotic relationship between *D. officinale* and mycorrhizal fungi is beneficial for *D. officinale*. RNA-Seq showed that endogenous hormones play a vital role in the seed germination of *D. officinale* (Wang et al., 2018). The relatively low concentrations of JA, abscisic acid (ABA) and strigolactones (SLs) may promote the growth of *D. officinale* (Wang et al., 2018; Chen J. et al., 2020). After inoculation of mycorrhizal fungi, genes associated with gibberellic acid (GA) biosynthesis were upregulated, and GA3 may be a key signaling molecule for germination (Chen J. et al., 2020). These results provide valuable insights for orchid-fungal symbiosis and seed germination in *Orchidaceae*. Under cadmium stress, *D. officinale* showed regulatory responses in roots through metal transporters, sulfate glutathione metabolism, cell wall metabolism, and phenylpropanoid metabolism (Jiang et al., 2020). Furthermore, as an epiphytic plant, *D. officinale* shows strong resistances to environments and can partly use crassulacean acid metabolism under stress conditions (Zhang et al., 2018; Zou et al., 2018). Although transcriptomic data on drought treatments have been obtained, further analysis still needs be performed to explore the mechanisms behind drought resistance (Wan et al., 2018; Zou et al., 2018).

As summarized above, many transcriptomic researches have been carried out in *D. officinale*, including various tissues, treatments or conditions. Tissue-specific patterns and key genes in the biosynthesis of bioactive compounds and responses to stresses have been extensively investigated. This research provides valuable molecular information for understanding these distinct physiological processes. At present, it is urgent to build a public, open database of transcriptional resources for *D. officinale* as the established database for *Arabidopsis thaliana* (The Arabidopsis Information Resource, TAIR), which can provide easy access to researchers, especially for those who understand the function of genes but are not familiar with bioinformatics tools. In the future, the newest sequencing techniques, especially the third generation platform, should be used for further analysis, such as alternative splicing.

## Proteomic Research in *Dendrobium Officinale*

Because the changes of gene expression profiles at the mRNA level are not always the same as the changes at the protein level, proteomic research in plants can help to reveal the molecular mechanisms of plant growth, development, metabolites and responses to environments. At present, the methods in proteomics include isobaric tags for relative and absolute quantification (iTRAQ), data-independent acquisition (DIA), and tandem mass tags (TMT), which can detect protein expression levels, post-translational modifications and protein-protein interactions of all proteins.

Proteomic research into *D. officinale* is a new avenue of research. The first report on *D. officinale* focused on analysis of root induced by *Mycena dendrobii* (Xu et al., 2015), which shows important proteins related to defense and stress responses, the formation of mycorrhizal fungi and the biosynthesis of bioactive components. These data explain why *M. dendrobii* can promote the growth of *D. officinale* seedlings. The lysine succinylation sites on *D. officinale* proteins were identified (Feng et al., 2017), and five key enzymes in the glycolytic pathway exhibit succinylation of lysine, which will promote understanding the functions of lysine succinylation in plants. Recently, to discriminate *D. officinale* from other *Dendrobium* species, among 343 measurable peptides, 29 peptides were chosen as putative biomarkers, and the short peptide ALGLELDLSER can be a biomarker for the identification of *D. officinale* plants from different geographical areas (Fang et al., 2020).

The aim of proteomics research in medicinal plants is usually to evaluate the active components and proteomes across different samples and dissect the molecular mechanisms of bioactive compounds. Compared with the gene expression profiles at the mRNA level, protein expression patterns can more reliably and accurately reflect the expression level of key enzymes in the biosynthesis pathways of active components. The deep research needs to be performed on the proteome of *D. officinale*, especially on the changes that occur at different stages and under various stresses.

## Metabolomic Research in *Dendrobium Officinale*

As metabolites represent the final products of gene expression and protein function, they can reflect changes in plant growth, development and responses to environments. Metabolomics has become a widely used tool in plant research, including wide-targeted metabolomics and targeted metabolomics. Due to bioactive functions of multiple metabolites in *D. officinale*, it is of great significance to analyze those metabolites (Table 3).

**TABLE 3:** A summary of metabolomic studies in *D. officinale.*

Materials	Treatments or conditions	Method	References
Stems	Three different substrates	UPLC-MS/MS	Zuo et al., 2020
Protocorm-like bodies	Precursors and methyl jasmonate	GC-MS, LC-MS	Jiao et al., 2018
Stems	Different growth years of two species	GC-MS	Jin et al., 2016
Stems	UV-B treatment	UPLC-MS/MS	Chen Y. et al., 2020
Caulis	Two *Dendrobium* plants	LC-MS	Song et al., 2020
Leaves and stems	–	LC-ESI-MS/MS	Cao et al., 2019
Stems	Different regions	GC–MS	Hu et al., 2020
			

The different metabolites in different *Dendrobium* species can be used to discriminate between them. There were 11 secondary metabolites that were considered as biomarkers of *D. officinale* and *Dendrobium huoshanense* (Jin et al., 2016). Moreover, in these two species, as many as 133 nitrogenous compounds were identified, and allyl alkaloid is an important medicinal component worth further investigation (Song et al., 2020). For *D. officinale* plants, the third year is the most suitable harvest time based on the accumulation of metabolites (Jin et al., 2016). The metabolites vary often in different tissues of *D. officinale*. The stems were composed of higher levels of multiple metabolites than the leaves, while leaves included higher concentrations levels of polyphenols and lipids (Cao et al., 2019). Moreover, 649 different metabolites were found in the stem and leaf of 6-month-old *D. officinale* (Cao et al., 2019), including organic acids, amino acids, nucleotides, and flavonoids.

Multiple factors influence the accumulation of metabolites. There were 101 volatile compounds detected in stems of *D. officinale* from four regions, and some distinctive compounds were found in certain region (Hu et al., 2020). UV-B radiation can induce accumulation of polysaccharides, alkaloids, and flavonoids in *D. officinale* stem (Chen Y. et al., 2020). The stems of *D. officinale* grown on different substrates contained significantly different metabolites (Zuo et al., 2020), and the mainly changed metabolites were the flavonoids. The stems from plants grown in pine bark showed a higher content of flavonoids, which provides the practical basis of substrate selection for *D. officinale* plants. Furthermore, the accumulation of alkaloids was increased when protocorm-like bodies of *D. officinale* were treated with tryptophan (secologanin) and MeJA (Jiao et al., 2018), and among them changes in 29 metabolites were confirmed, including carapanaubine, a kind of TIAs.

To date, metabolomics studies into *D. officinale* remain limited. However, with technological development, more attention could be placed on this area because it is home to important medicinal properties in need of elucidation. Metabolomics studies not only provide us insights of metabolite accumulation under different conditions, but also important potential management methods that can improve the contents of target metabolites. Moreover, although wide-targeted metabolomics can provide huge information regarding metabolites, more focuses needs be paid to the active compounds through targeted metabolomics in *D. officinale*, which can provide more accurate information for certain type of metabolites.

## Integration Research of Multi-Omics in *Dendrobium Officinale*

The process of plant growth and development is complex and changeable. The plant status is the result of interactions between the genetic information and environmental factors. Integration of multi-omics can provide more comprehensive information to understand the dynamic changes and the potential mechanisms of various physiological processes. At present, there are some reports on the integration of different omics in *D. officinale* as shown in Table 4. Mainly, transcriptomics and metabolomics are associated to dissect changes in gene expression and metabolite accumulation in different tissues or under special conditions, which can be used to construct a regulatory network.

**TABLE 4:** A summary of omics integration in *D. officinale.*

Samples	Treatments or conditions	Combinations of omics	Research focus	References
Stems	Purple and normal varieties	UPLC-ESI-MS/MS, RNA-seq	Pigmentation	Zhan et al., 2020
Flowers	Buds and opened flower	RNA-seq, HPLC-MS	Flower development	He et al., 2020
Seeds	Asymbiotic and symbiotic at different stages	RNA-Seq, iTRAQ	Symbiotic germination	Chen J. et al., 2017
Leaves	at 0°C and at 20°C	GC-MS, RNA-seq	Cold acclimation	Wu et al., 2016
Leaves	NaCl treatment	RNA-seq, LC–MS/MS	Salt stress	Zhang et al., 2021
Stems	Fungus MF23	RNA-seq, UHPLC-LTQ	Fungus on growth	Shan et al., 2021
Flowers	Two cultivars	RNA-seq, GC-MS	Volatile terpenoids	Li N. et al., 2021

The combination of metabolomic and transcriptomic analysis has also widely been used to analyze various physiological processes in *D. officinale*, such as flower development (He et al., 2020), cold acclimation (Wu et al., 2016), stem color (Zhan et al., 2020), salt stress (Zhang et al., 2021), and flower volatile terpenoids (Li N. et al., 2021). Some key factors have been identified to explain why the metabolites were changed, including the key enzymes in the biosynthetic pathway and potential transcription factors (Li N. et al., 2021). For example, the increase in content of delphinidin and quercetin derivatives was responsible for the purple stem of *D. officinale* and *F3H* and leucoanthocyanidin dioxygenase (*LDOX*) genes were found to be highly expressed (Zhan et al., 2020). Through analysis of seed germination by transcriptomics and proteomics (Chen J. et al., 2017), 32 genes showed consistent levels at mRNA and protein level and some of them were related to lipid and glycometabolism, which may be induced by fungi during germination, offering valuable insights into seed germination for orchid plants. *Mycena* sp. can promote the growth of *D. officinale* due to better nitrogen uptake and NH_4_^+^ assimilation (Shan et al., 2021), which supplies the molecular basis for cultivation.

Reports on combination of different omics are presently limited in *D. officinale*. However, the ample advantages of the approach are amply demonstrated (Table 4). The molecular status of plants can comprehensively be reflected at different levels, which is conducive to screening for trait-related genes and for further investigation.

## Perspectives

Due to its high medicinal and economic values, *D. officinale* has attracted significant attention from researchers. Thanks to the rapid development of sequencing and analysis methods, close relationships have been built between traditional Chinese medicine and genetic basis (Tables 1–3). Various biological processes of medicinal plants including *D. officinale* are now increasingly being investigated. Among them, the omics methods have been considered as a powerful tool to obtain a huge amount of information, including gene sequences, gene expression patterns, protein expression profiles, and metabolites in *D. officinale*. Therefore, in-depth studies of the molecular mechanisms underlying the biological processes are now possible and credible, such as the accumulation of active components. In recent years, many breakthroughs have been made in the study of *D. officinale*. Of particular note, the third version of the *D. officinale* genome at the chromosome level has recently been reported (Niu et al., 2021), which facilitates future genomic research. High-quality genomic data of *D. officinale* can have a vital influence on further studies into the accumulation and regulation of active components and molecular breeding of *D. officinale*. Transcriptomics of *D. officinale* is mainly focused on the key enzyme genes in the pathway of active component synthesis and analyzing its metabolic pathway.

Although great strides have been made in deepening our understanding of *D. officinale*, limitations remain. First, many genes related to active components in *D. officinale* have been identified by genomic and transcriptomic studies (Figures 1–3), but the functions of most these genes have not been confirmed through functional investigation, and their regulatory mechanisms have not yet been elucidated. For example, MeJA has been shown to induce the accumulation of polysaccharides, flavonoids, and alkaloids in *D. officinale* (Yuan et al., 2017; Jiao et al., 2018; Chen et al., 2019; Adejobi et al., 2021). However, the key factors in the JA signal pathway still need to be identified and clarified. Second, *D. officinale* plants generally live in extremely harsh environments and show strong drought resistance, the molecular mechanism of which is still limitedly clarified. There is a large amount of transcriptomic data available on *D. officinale* treated under different drought conditions (Wan et al., 2018; Zou et al., 2018). However, these data have not yet been fully and deeply analyzed. Therefore, there is an urgent need to excavate the data to reveal the molecular mechanisms of drought resistance in *D. officinale*.

**FIGURE 4:**
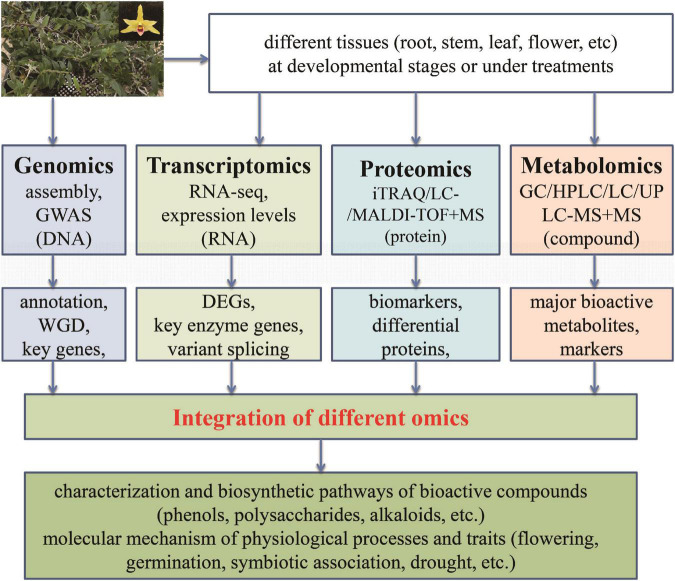
Application of multiple omics in *Dendrobium officinale*. The integration of data from different levels (DNA, RNA, protein, and metabolites) can make it feasible and reliable to discover the potentially key factors (transcription factor, gene, protein, or compound) responsible for bioactive compounds as well as physiological processes and traits.

In order to promote further researches into *D. officinale*, attentions should be paid to the following work in the future: (1) Although current omics technologies have been used to study *D. officinale*, there are limitedly comprehensive multi-omics studies. Obviously, one single omics method such as transcriptomics, proteomics, or metabolomics cannot satisfy the necessary deep research into *D. officinale*. Therefore, it is imperative to study the mechanisms that underlie biosynthesis and the regulation of active compounds in *D. officinale via* the integration of multi-omics (Figure 4). In particular, the combination of proteomics and metabolomics has not yet been carried out for *D. officinale*. (2) The genuine resource of medicinal plants is essential for their functions, including *D. officinale*. Therefore, it is important to comprehensively investigate why differences exist in active compounds between plants from different cultivation regions or conditions at the genome, mRNA and protein levels, which can provide propitiate conditions for metabolite accumulation. (3) Based on the whole genome of *D. officinale*, resequencing of different germplasm resources can help to screen the key genes responsible for interesting traits for further functional dissection. In particular, studies on the mechanisms of biosynthesis and regulation of growth and development of active ingredients should be continued. (4) Although some genes belonging to the biosynthetic pathway of the main active ingredients in *D. officinale* have been identified, the accurate functions of only a few genes have been confirmed. Studies on key genes related to the biosynthesis of active components, growth regulation, and stress resistance of *D. officinale* require further investigated. Recently, the potential transformation system for *Dendrobium* plants was reported (Li Y. et al., 2021), which could benefit the functional investigation into *D. officinale*.

## Author Contributions

AL: conceptualization and writing – review and revising. YueW, OA, YT, and YuhW: literature search and data analysis. YueW: writing – original draft. All authors have read and agreed to the published version of the manuscript.

## Conflict of Interest

The authors declare that the research was conducted in the absence of any commercial or financial relationships that could be construed as a potential conflict of interest.

## Publisher’s Note

All claims expressed in this article are solely those of the authors and do not necessarily represent those of their affiliated organizations, or those of the publisher, the editors and the reviewers. Any product that may be evaluated in this article, or claim that may be made by its manufacturer, is not guaranteed or endorsed by the publisher.
